# Head and Neck Cancer in Fanconi Anemia: Clinical Challenges and Molecular Insights into a DNA Repair Disorder

**DOI:** 10.3390/cancers17183046

**Published:** 2025-09-18

**Authors:** Juhye Choi, Moonjung Jung

**Affiliations:** 1 Faculty of Medicine, Semmelweis University, 1085 Budapest, Hungary; 2 Division of Hematology, Department of Medicine, The Johns Hopkins University School of Medicine, Baltimore, MD 21205, USA; 3 Department of Oncology, Sidney Kimmel Comprehensive Cancer Research Center, The Johns Hopkins University School of Medicine, Baltimore, MD 21205, USA; 4 Department of Genetic Medicine, The Johns Hopkins University School of Medicine, Baltimore, MD 21205, USA

**Keywords:** Fanconi anemia, head and neck cancer, HNSCC, germline predisposition, germline pathogenic variant

## Abstract

Fanconi anemia (FA) is a genetic disorder characterized by congenital anomalies, bone marrow failure, and cancer predisposition. Among other solid cancers, head and neck squamous cell carcinoma (FA HNSCC) is the most common cancer type in individuals with FA. The FA pathway is required for the complete repair of DNA interstrand crosslinks (ICLs), and unresolved ICLs result in cell cycle arrest, apoptosis, or complex chromosomal rearrangements due to chromosome breaks, ultimately leading to tumorigenesis. FA HNSCCs present earlier (median age of onset in the 30s) and exhibit a more aggressive course with frequent recurrence and second primaries, and entail a poorer survival rate compared to sporadic HNSCC. FA HNSCCs are mostly human papillomavirus (HPV)-negative and frequently carry somatic copy number variations (CNVs), which amplify oncogenes implicated in sporadic HNSCC, but single-nucleotide variants or small insertions and deletions are less frequent than in HPV-negative sporadic HNSCC. A subset of sporadic HNSCC carries pathogenic mutations or promoter methylation in FA genes, which also harbor characteristic somatic CNVs, suggesting shared molecular underpinnings with FA HNSCC. Heightened inflammation from genomic instability and transcriptional activation of retrotransposons contribute to tumorigenesis and increased invasiveness by the epithelial-to-mesenchymal transition. Due to heightened sensitivity to DNA crosslinking agents in patients with FA, platinum-based chemotherapy is generally avoided, which presents a significant hurdle for treatment and thereby leaves limited therapeutic options. Surgical management is the mainstay of therapy if possible, and targeted therapy has been increasingly studied in HNSCC in FA.

## Simple Summary

Fanconi anemia is an inherited bone marrow failure syndrome with an increased incidence of cancers due to DNA repair deficiency. Head and neck cancer is the most common solid cancer type in patients with Fanconi anemia. In this review article, we summarize clinical manifestations and discuss recent discoveries in the molecular pathogenesis of tumor formation and progression in DNA repair disorder, Fanconi anemia. We also discuss that a subset of head and neck cancers in the general population carries genetic or epigenetic alterations, impacting the Fanconi anemia pathway, and they share molecular features similar to those associated with Fanconi anemia, highlighting the importance of the Fanconi anemia DNA repair pathway in the prevention of head and neck cancer.

## 1. Introduction

Fanconi anemia (FA) is a largely autosomal recessive inherited bone marrow failure syndrome, except for FA complementation group B (*FANCB*), which is X-linked recessive, and *FANCR/RAD51*, which exhibits autosomal dominant cellular effects [1,2,3,4]. FA is characterized by congenital anomalies affecting major organ systems, bone marrow failure (BMF), and an extreme predisposition to malignancies [1]. Individuals with FA have an exquisitely increased risk of head and neck squamous cell carcinoma [5,6,7], here termed FA HNSCC. While FA is a rare genetic disorder, studying the pathogenesis of FA allows us to develop a deeper understanding of the interplay of DNA repair pathways and tumorigenesis, which can inform the development of novel diagnostic and therapeutic strategies for cancers in the general population. In this review article, we summarize clinical manifestations of FA with a focus on FA HNSCC, critically review the recent basic/translational original research papers investigating molecular underpinnings of extreme cancer predisposition, and briefly discuss the current treatment paradigm.

## 2. Clinical Manifestations of FA

### 2.1. Congenital Anomalies, BMF, Leukemia

FA manifests significant genetic and phenotypic heterogeneity [8], occurring in approximately 1 in 100,000 to 1 in 160,000 live births, with a carrier frequency of 1 in 181 in North America [9,10,11]. Mutations in at least 23 genes have been implicated in its defective DNA repair pathomechanism [1], and clinical manifestations may vary significantly [12]. About three-quarters of FA patients present with a spectrum of congenital malformations ranging from cafe-au-lait spots (55%), short stature (51%), and limb defects (43%) to anomalies in the head (26%), eyes (23%), and kidneys (21%) [1,13]. Although the remaining one-quarter of patients do not exhibit congenital anomalies, they frequently demonstrate stunted growth below the fifth percentile and are associated with endocrine abnormalities such as insulin resistance and growth hormone deficiency [9].

Blood count is typically normal at birth, yet macrocytosis soon ensues, followed by thrombocytopenia and anemia. Hematologic aberrations can be the first presenting symptoms of FA in those patients without congenital anomalies and are considered to be the most predominant clinical features responsible for marked morbidity and mortality [14,15]. Notably, pancytopenia arises at a median age of 7 [14] with the cumulative incidence of bone marrow failure (BMF) culminating in 90% by age 40 [16]. A significant number of patients further progress to develop myelodysplastic syndrome (MDS) and/or acute myeloid leukemia (AML) [17] as well as solid tumors, most commonly squamous cell carcinomas of the head and neck, and anogenital region. Such heightened cancer susceptibility can be explained by the high degree of chromosomal instability that arises from the defective DNA crosslink repair mechanism [16], thereby prompting early surveillance and timely intervention, including crucial hematopoietic stem cell transplantation (HSCT). Altogether, FA presents a complex clinical vignette of developmental, hematopoietic, and carcinogenic phenotypes [18]. The projected median survival for patients is approximately 30–40 years, although this may vary depending on various compounding factors such as the severity of BMF, congenital anomalies, HSCT, and malignancies [13,19].

### 2.2. Clinical Characteristics of FA Cancers

FA implicates a dramatically increased risk for both hematologic and solid malignancies, thus rendering patients vulnerable to early-onset, aggressive cancers that have a significant impact on their overall survival. Observational studies estimate an observed-to-expected ratio for cancer in FA to be approximately 50-fold overall, including 48-fold for solid tumors and up to 800-fold for hematologic malignancies [5]. FA-associated MDS shows an exceptionally high 5500-fold risk [20], manifesting as refractory cytopenia with multilineage dysplasia and progressing to AML in 9% of cases [21,22]. Hazard for AML rises after age 10 and plateaus by age 30. While HSCT can prevent both MDS and AML if performed early in the course, it does not prevent the development of solid tumors and may even increase the carcinogenic risk due to persistent genomic instability, increased age of the patient, and graft-versus-host disease (GVHD) [23].

Among the solid tumors that arise in the context of FA, the occurrence of HNSCC is exceptionally high. FA is associated with a 500- to 700-fold elevated risk of HNSCC. Based on the clinical data in the International Fanconi Anemia Registry (IFAR), FA patients develop HNSCC at a median age of 31 (range: 15–49), which is significantly younger than the typical median age of onset of 66 years in HPV-negative HNSCC and 53 years in HPV-positive HNSCC [16,24,25]. Furthermore, they also have shorter cancer-specific survival (median survival 17 months) [24]. Pathogenic variants of the *FANCA* gene are the most common among FA patients. *FANCA* exon 27–30 variants are specifically involved in the development of HNSCC and gynecological malignancies [26]. Pathogenic variants in the *FANCA*, *FANCC*, and *FANCG* genes make up the majority (up to 90%) of FA cancer cases, which is similar to the FA genotype distribution [27]. The most common site for HNSCC is the oral cavity, primarily the tongue and the buccal mucosa, but tumors can arise in the pharynx and the larynx as well [21]. Like other FA-associated cancers, FA HNSCC tends to be clinically aggressive, and secondary primary malignancies are also common. Patients’ five-year overall survival rate hovers around 39%, with locoregional recurrence exceeding 50% within 16 months [6]. More than 60% of patients develop second tumors [23]. The primary treatment modality for FA HNSCC is surgery, but the outcome is usually poor as patients are often diagnosed at advanced stages. Treatment becomes even more challenging as many patients experience toxicity-related adverse events (e.g., severe mucositis and prolonged pancytopenia) that arise from hypersensitivity to DNA crosslinking agents, which severely constrain the use of adjunct radio- and chemotherapy [28]. Therefore, in order to improve cancer outcome in FA HNSCC, early detection by regular screening is critically important.

### 2.3. Genotype–Phenotype Correlation in FA Cancers

Most individuals with FA carry biallelic mutations in *FANCA*, followed by *FANCC* and *FANCG*. They generally follow the clinical patterns of BMF, MDS/AML, and solid tumors, most commonly HNSCC, as described above. Several hypomorphic variants have been reported in the literature (Table 1), which modify the clinical presentation. Patients who carry at least one allele of a hypomorphic variant commonly present with milder BMF and a delayed onset of solid tumors. Sometimes, it may be difficult to distinguish whether the patients carry hypomorphic variants or somatic reversion (also known as somatic mosaicism or somatic genetic rescue), which requires functional assays or sequencing analysis to differentiate.

Individuals with biallelic pathogenic variants in *FANCD1/BRCA2* and *FANCN/PALB2* exhibit a unique cancer predisposition [40,41]. Brain tumors (mostly medulloblastoma), Wilms tumor, and neuroblastoma are almost exclusively seen in patients with *FANCD1/BRCA2* and *FANCN/PALB2* variants [41]. These embryonal tumors generally occur within two decades of life, with Wilms tumor and neuroblastoma occurring exclusively within 5 years of life [41,42]. AML is also frequent, as in the rest of the FA groups, but it tends to occur at a younger age, primarily below age 5 [22].

Individuals with biallelic mutations in *FANCM* usually do not develop classical FA (i.e., no congenital anomalies or BMF) despite positive chromosome breakage testing [43,44]. Rather, they develop early-onset cancers (breast, HNSCC, acute leukemia) and premature gonadal failure [43,44,45,46]. While they do not present with BMF, they often experience severe chemotherapy toxicity [44,47]. Given these clinical differences, the ClinGen Gene Curation Expert Panel newly classified FANCM Fanconi-like genomic instability disorder separately from FA [48].

Overall, these genotype–phenotype relationships serve to demonstrate the heterogeneous clinical presentations of FA-related cancers and further allude to the need for genotype-specific surveillance and treatment strategies.

### 2.4. Increased Risk of FA HNSCC Post-HSCT

While curative for FA-induced BMF, HSCT increases the risk for SCC by 4.4-fold. Interestingly, the peak age at which MDS/AML occurs is 20 years, after which the occurrence rate of hematologic events stabilizes or decreases. This is in contrast to the risk of solid tumor development, which remains relatively low prior to age 20 and subsequently increases thereafter [5]. In fact, approximately 76% of FA patients are likely to have developed a solid tumor by the age of 45 [28]. Such patterns may indicate that HSCT contributes to solid tumor development. Alternatively, it may imply that the age-associated accumulation of DNA lesions plays a critical role in tumorigenesis, and we are seeing an increasing incidence of solid cancers as FA patients are now more likely to survive into their adulthood thanks to successful HSCT.

The association between HSCT and SCC is a multifaceted one that involves chronic immunosuppression, mucosal injury, and DNA repair deficiencies. Most predominantly, the pre-transplant conditioning regimen employs alkylating agents and radiotherapy, both of which introduce massive DNA damage and overwhelm the already-impaired DNA repair mechanism in FA patients [18]. Additionally, GVHD that occurs post-HSCT can lead to sustained epithelial injury and inflammation, thereby creating a pro-tumorigenic environment [49]. Together, these insults accelerate carcinogenesis in post-transplant FA patients. In particular, clinical evidence shows that patients with grade III/IV acute or chronic GVHD have significantly increased SCC incidence [12], and SCC in this setting tends to be multifocal, often presenting with earlier onset and faster progression than in non-transplant FA patients [26]. Radiation and chemotherapy are poorly tolerated [50], thereby rendering surgery the principal treatment method. However, survival is limited, with a median post-SCC survival of six months, and the long-term outcome remains poor [51].

## 3. Molecular Pathogenesis of FA

### 3.1. Fanconi Anemia DNA Repair Pathway

The FA pathway consists of at least 23 genes, *FANCA* through *FANCX*, which are required to repair DNA interstrand crosslinks (ICLs) [52,53,54,55,56]. Exogenous DNA crosslinking agents, such as mitomycin C or cisplatin, or endogenous reactive aldehydes such as acetaldehyde and formaldehyde, cause ICLs. ICLs are covalent bonds between the Watson and Crick DNA strands, and their presence prevents the separation of DNA strands and interferes with replication and transcription [57]. Therefore, unrepaired ICLs are detrimental to cells, leading to p21–p53 activation and apoptosis [58].

When ICLs arise from endogenous or exogenous crosslinking agents, FANCM and FA-associated protein 24 (FAAP24) initially recognize the presence of ICLs during the S phase, activate the ATR-Chk1-dependent DNA damage response, and recruit the FA complex components, including FANCA, FANCB, FANCC, FANCE, FANCF, FANCG, FANCL, and FANCX (encoded by the most recently reported FA gene; also known as FAAP100) [55,56,59,60]. The FANCB-FANCL-FANCX subunit (the E3 ubiquitin ligase subunit), along with FANCT (E2 ubiquitin conjugating enzyme) [61], monoubiquitinates FANCD2 and FANCI. Monoubiquitination of FANCD2 and FANCI functions as a clamp for the FANCI-FANCD2 (ID2) complex, so that it can dock at the site of ICL repair and recruit downstream repair proteins [60]. Nucleases, including FANCP/SLX4 and FANCQ/XPF, make an incision on either side of ICLs, also known as “unhooking”, allowing translesion polymerases, such as pol zeta (subunit FANCV), to repair one DNA strand [62,63,64]. Then, homologous recombination factors, such as FANCD1/BRCA2, FANCS/BRCA1, FANCN/PALB2, FANCR/RAD50, FANCJ/BRIP1, FANCO/RAD51C, FANCW/RFWD3, and FANCU/XRCC2, repair double-stranded DNA [65]. Loss-of-function mutations in any one of 23 FA genes lead to the disease, Fanconi anemia.

### 3.2. Molecular Mechanisms of FA Tumorigenesis

The large-scale sequencing study revealed that FA SCCs (mostly head, neck, and esophageal origin) are characterized by a high number of structural variants, most commonly small deletions, unbalanced translocations, and fold-back inversions [24]. Structural variants are often connected to each other, resulting in complex chromosomal rearrangements, which arise in the background of loss-of-function mutations in *TP53* [24]. *TP53* mutation allelic frequency was 85%, which suggests that loss of p53 is an early event in tumorigenesis, and it allows additional genetic alterations to occur without DNA-damage-induced apoptosis [24]. Interestingly, human papillomavirus (HPV)-positive tumors were rare in FA SCC, suggesting a different underlying mechanism of tumorigenesis between HPV-positive sporadic HNSCC and FA SCC. HPV-positive and -negative sporadic HNSCC are considered clinically and molecularly distinct entities, with HPV-negative tumors exhibiting a poorer response to therapy, worse prognosis, and higher mutation burdens compared to HPV-positive tumors. Indeed, FA SCCs bear a greater resemblance to HPV-negative tumors than to HPV-positive ones, albeit with distinct clinical and genomic features. Molecular differences were, for example, single-nucleotide variants (SNVs) and small insertions and deletions (indels) being less frequent in FA SCC than in HPV-negative sporadic HNSCC. On the other hand, somatic CNVs were much more frequent in FA SCCs, perturbing oncogenes and tumor suppressor genes implicated in HNSCC by amplification or deletion, respectively. Clinically, smoking and alcohol exposure are the main risk factors for HPV-negative sporadic HNSCCs. In contrast, FA SCCs arise in the absence of those risk factors due to inherent DNA repair defects. Basal levels of endogenous aldehydes from normal cellular metabolism are likely to be enough to cause chromosome breakage in FA, while higher levels of aldehydes from smoking and alcohol drinking are required in the general population to develop HNSCC.

### 3.3. Sporadic HNSCC and Somatic Alterations in the FA Pathway

The deficient FA pathway not only plays a crucial role in tumorigenesis in FA but also contributes to tumorigenesis in sporadic HNSCC. A subset of sporadic HNSCC with altered FA genes shows genomic changes more similar to FA HNSCC [24]. A prior study performed quantitative RT-PCR on 49 consecutive primary sporadic oral SCC samples. It showed that 66% showed at least 5-fold downregulation, and 18% showed greater than 40-fold downregulation of one of ten FA genes tested [66]. Among them, downregulation of *FANCB, FANCF, FANCJ*, and *FANCM* was most notable. While the level of downregulation required to abrogate the FA pathway function is unknown, this study shed light on the mechanistic link between the FA pathway and sporadic HNSCC [66]. Profiling 18 sporadic HNSCC cell lines revealed that 53% of them showed increased chromosomal breakage with exposure to mitomycin C or cisplatin, ICL-generating agents, which is a pathognomonic feature of FA, albeit only a subset of them had identifiable pathogenic mutations in FA genes or promoter hypermethylation of *FANCF* [67]. Another study showed that about one-third of patient samples with sporadic HNSCC (14/45) showed promoter hypermethylation in *FANCB* [68]. More recent re-analysis of TCGA dataset showed that up to 13% of HPV-negative sporadic HNSCC cases harbored deletions in *FANCV/MAD2L2*, *FANCR/RAD51*, *FANCU/XRCC2*, as well as *ALDH2*, a known FA disease modifier. These tumors exhibited a higher frequency of somatic CNV compared with the complete HPV-negative sporadic HNSCC cohort [24]. These findings suggest that the proficient FA pathway is critical in preventing sporadic HNSCC, and cells with the somatically altered FA pathway may be more vulnerable to DNA crosslinking agents.

### 3.4. Fanconi Anemia Pathway and the Health of Epithelial Cells

Sensitivity to DNA crosslinking agents leading to chromosomal breakage is the hallmark of FA. Chromosomal breakage also occurs in buccal epithelium [69], and underlying genomic instability is the major driver of SCCs [70]. Micronuclei testing in buccal epithelium has been proposed as a biomarker to assess therapy responses [69]. Surprisingly, individuals with FA have other defects in the epithelial structures beyond the underlying genomic instability. For example, epidermal organoid differentiation from patient-derived iPSC revealed that the FA pathway is required for proper adhesion and controlled basal cell proliferation [71]. Epidermal junction molecules required for epidermal integrity, such as desmosomes and hemidesmosomes, were reduced in number and structurally abnormal, leading to increased blistering in individuals with FA compared with healthy controls [71]. These abnormalities may further exacerbate tissue injury and inflammation, contributing to tumorigenesis and tumor progression.

### 3.5. Epithelial-to-Mesenchymal Transition (EMT) and Heightened Pro-Inflammatory Signaling

Epithelial-to-mesenchymal transition (EMT) is a phenomenon in which epithelial cells acquire mesenchymal phenotypes [72]. EMT in cancer is characterized by the induction of EMT-related gene expression, allowing cytoskeleton changes and increased mobility of cancer cells and basement membrane invasion [72]. EMT is also associated with poor prognosis and therapy resistance in HPV-negative HNSCC [72,73]. Isogenic HNSCC cell lines with and without a proficient FA pathway showed that the loss of the FA gene function resulted in an increase in vimentin expression, intercellular projections, and invasiveness, which was associated with increased activities of DNA-dependent protein kinase (DNA-PK) and Rac1 GTPase [74], suggesting that the FA pathway is required to maintain epithelial cell morphology with intact cellular organization. In a mouse serial allograft model of FA SCC, *Fanca*−/− keratinocytes showed earlier EMT than *Fanca+/+* keratinocytes [24]. Primary human FA SCC samples also showed a strong partial-EMT gene expression profile [24]. DNA-damage-induced inflammatory signaling, particularly non-canonical NF-kB pathway activation, was postulated to accelerate EMT in FA SCCs [24,75]. Additionally, SLX4 plays a role in maintaining repression of transposable elements, such as LINE1. For example, the SLX4-MUS81-EME1 complex is required to repress LINE1 retrotransposition, and *SLX4*-null cells showed increased cytoplasmic DNA, cGAS-STING pathway activation, and heightened interferon signaling [76]. This pro-inflammatory signaling could also, in turn, promote EMT [77].

## 4. Management of FA Cancers

### 4.1. Cancer Screening Recommendations for FA

Due to the markedly elevated cancer risk in FA, proactive and preferably genotype-tailored cancer screening is critical. Current guidelines recommend complete blood count screening every 3–6 months and annual bone marrow evaluations to monitor for hematologic abnormalities [78]. In patients with *FANCD1/BRCA2* or *FANCN/PALB2* variants, additional screenings for embryonal tumors (e.g., brain MRI, abdominal ultrasound, and urine catecholamine) are recommended [41,78].

Considering the high, early incidence of HNSCC post-HSCT, patients are advised to undergo biannual oral examinations starting early childhood in addition to annual nasopharyngolaryngoscopy [79]. In case suspicious lesions are found, a biopsy is performed to analyze the morphology and the chromosomal ploidy. While the gold-standard approach for a suspicious lesion is an incisional or excisional biopsy, given the frequent occurrence of invasive procedures in patients with FA, it is associated with decreased quality of life and reduced adherence to screening. To improve the quality of life in these patients while maintaining high sensitivity and specificity, several non-invasive screening methods have been investigated (see the review article by Beddok et al. [80] for a detailed review of screening strategies). Velleuer et al. employed oral brush biopsy cytology to assess at least 737 FA HNSCC lesions, and the procedure alone achieved 97.7% sensitivity and 84.5% specificity. The addition of aneuploidy analysis to cytology samples further increased the sensitivity to 100% and specificity to 92.2% [81]. Moreover, incorporating machine learning can enhance the accuracy of conventional cytology by reducing the subjectivity of the pathologist [82]. Furthermore, next-generation sequencing (NGS) has recently been incorporated into brush cytology samples, which may aid in the earlier detection of high-risk pre-cancerous lesions as well as provide prognostic information that can be used for follow-up and treatment decisions [83]. The external validation and broader implementation of these newer non-invasive screening techniques have great potential to improve the screening outcome of FA HNSCC.

### 4.2. Overview of Current and Future Treatment for FA HNSCC

Given the pathomechanistic background of DNA repair defects, FA patients often demonstrate poor tolerance to the conventional genotoxic therapies. DNA-damaging chemotherapy and/or radiation therapy frequently entail severe and sometimes life-threatening toxicities, most commonly high-grade mucositis, dysphagia, and prolonged cytopenias [6,84]. Consequently, surgical intervention serves as the main pillar for FA HNSCC treatment; with early detection, surgical resection can provide durable control of the disease and sometimes even be curative (see Lee et al. [84] for a systematic review of the treatment of FA HNSCC). While adjuvant systemic platinum-based chemotherapy, such as cisplatin, is generally avoided due to excessive toxicities, reduced-dose radiotherapy with careful monitoring has proven to be both feasible and tolerable in many cases [84].

In light of such limitations on conventional therapies, several alternatives with lower genotoxicity, including targeted agents and immunotherapies, have recently emerged and shown promising evidence. A preclinical study that performed a high-throughput screen of 3802 drugs has identified small molecules that inhibit epidermal growth factor receptor (EGFR) as top candidates for targeting *FANCA*-deficient HNSCC [85]. According to this study, EGFR inhibitors such as gefitinib and afatinib demonstrated high tumor-to-non-tumor IC50 ratios (approximately 400 and 100, respectively) *in vitro* and suppressed tumor growth in xenograft *FANCA*-HNSCC *in vivo* models without inducing significant hematopoietic toxicity in *FANCA*-deficient mice. Such data, though derived from the preclinical stage, have shown sufficient promise that these candidates have been granted orphan drug designation by the European Medicines Agency [85], and the relevant clinical trials are ongoing. This avenue of treatment renders tumor NGS a particularly crucial component of the treatment decision, as it may enable the identification of actionable pathways to guide the selection of targeted therapies, which are usually better tolerated in patients with FA than conventional chemoradiation therapy.

Immune checkpoint inhibitors (ICIs), such as anti-PD-1/PD-L1 antibodies or anti-CTLA4 antibodies, are another potential pillar of systemic therapy for advanced HNSCC, though with limited clinical evidence. A systematic review of approximately 120 FA HNSCC cases that have occurred over 1966–2020 shows that immunotherapy has been employed as part of the treatment regimen in select cases. Given the potential risk of flaring of acute and chronic GVHD in post-transplant patients, ICI is cautiously used for the transplant population, showing mixed results [84]. The tolerability and efficacy of ICI in FA HNSCC need to be studied in prospective clinical trials.

On the whole, preferable early detection and surgical resection remain the primary treatment modality for FA HNSCC due to the severe toxicity constraint imposed by the conventional chemo- and radiotherapy. Adjuvant therapy is thus utilized with great caution after careful balancing of the toxicity risks with local control of the tumor. Although still in developmental stages, targeted and immune-based therapies are emerging as a new alternative therapeutic paradigm that aims for the precise, highly selective destruction of FA cancer cells while inflicting a tolerably low level of damage to other tissues. Ultimately, the search for the optimal agents in specific combinations, dosages, and sequencing for particular patient subsets is key to improving outcomes in FA HNSCC patients.

## 5. Discussion

**Figure 1:**
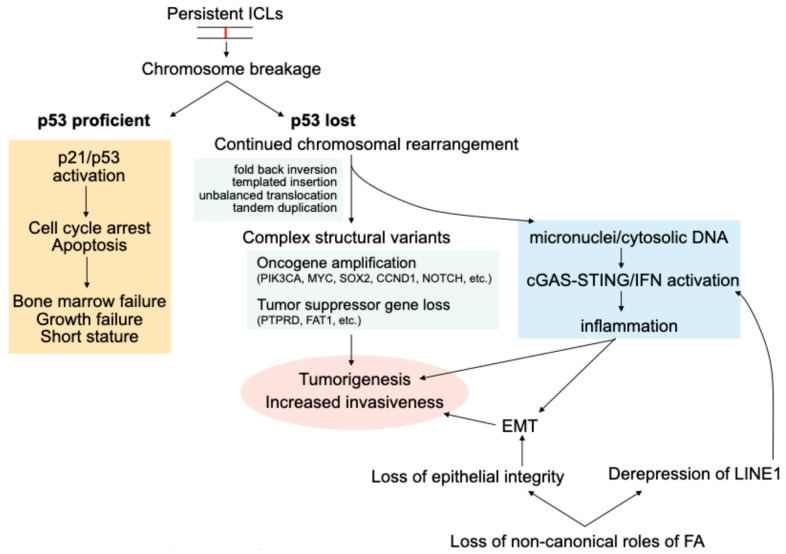
Overview of the pathogenesis of FA HNSCC. Persistent ICLs may lead to chromosomal breakage and complex rearrangements. The loss of p53 is generally an early event, allowing for complex genomic alterations without apoptosis. Complex structural variants involving oncogenes or tumor suppressor genes promote tumorigenesis. Heightened inflammation and intrinsic defects in epithelial integrity accelerate EMT, contributing to the aggressiveness of FA HNSCC. Arrows indicate sequential events and/or causal relationships.

We reviewed the latest basic and translational original research papers to deepen our understanding of the molecular pathogenesis of FA HNSCC. Understanding the basic mechanism of FA HNSCC enables us to develop novel therapeutic strategies not only for FA HNSCC but also sporadic cancers, as the FA DNA repair pathway is the critical genome guardian against human cancers. The inability to repair ICLs in FA results in an extreme cancer predisposition [86]. Endogenous and exogenous aldehydes and other crosslinking molecules from alcohol, smoking, or chemotherapeutic agents are important causes of ICLs in FA [87,88]. Unresolved ICLs lead to a hyperactive p53 axis and apoptosis, leading to bone marrow failure and poor growth [58]. Loss of the p53 axis appears to be a prerequisite for the development of FA HNSCC, given the high variant allelic frequency of *TP53*, suggesting an early event [24]. Chromosomal breakage caused by crosslinking molecules, pathognomonic of FA, leads to complex rearrangements, which result in characteristic somatic CNV conferring a survival advantage and resistance to apoptosis due to oncogene amplification [24] (Figure 1). Because the molecular defect in FA is the failure of ICL repair resulting in chromosome breakage, complex structural abnormalities are frequent, while SNVs are in fact less frequent than sporadic HNSCC [24]. A subset of HNSCC in the general population harbors pathogenic mutations or promoter hypermethylation in FA genes, which shows similar genomic alterations to FA HNSCC, highlighting the critical role of the FA DNA repair pathway in the genome maintenance of epithelial cells. While we have discovered complex genomic features of FA HNSCC by large-scale sequencing studies, safe and effective systemic treatment for this deadly disease is still unavailable. Given the poor tolerability of conventional chemoradiation therapy in patients with FA, we need to continue to find novel non-genotoxic therapeutic strategies, such as patient-specific molecular targeting of tumors. Early detection and complete surgical resection are the most effective strategies for improving outcomes in FA HNSCC to date. Given that the oral cavity, especially the tongue, is the most frequent site, regular surveillance, including frequent self-examination, is recommended. Recently developed oral brush biopsy cytology, combined with machine-learning-based automated microscopy, aneuploidy analysis, and/or tumor NGS, has the potential to improve the early detection rate of FA HNSCC while preserving the superior quality of life, leading to increased screening adherence [80].

As an adult oncologist, one may encounter adult patients who have not been diagnosed with FA, but HNSCC is the initial presenting manifestation of FA. For example, if a young patient without a significant alcohol or smoking history presents with HNSCC, or patients develop severe mucositis or profound cytopenia out of proportion after just one cycle of chemotherapy, one should raise a suspicion for FA. Peripheral blood chromosome breakage testing is the diagnostic test of choice for FA; however, if pre-chemotherapy blood counts were normal, then it may represent somatic reversion. Therefore, chromosome breakage testing should be performed on skin fibroblasts in select patients with normal peripheral blood chromosomal breakage testing but with a high clinical suspicion of the index.

## 6. Limitations

Our review article focuses on summarizing the latest basic and scientific original research on FA HNSCC and providing a general high-level overview of current practice among FA experts. FA is a rare genetic disorder; therefore, large-scale randomized controlled trials specifically studying this rare disorder do not exist, and patients with FA are often excluded from clinical trials for sporadic HNSCC. Therefore, high-quality clinical evidence is generally lacking in the FA HNSCC space. As such, our review on the management of FA HNSCC (e.g., screening and treatment) is mainly based on small case series with limited strength of evidence.

## Figures and Tables

**Table 1:** Hypomorphic variants reported in the literature are associated with milder FA phenotypes.

Gene	Variant	Clinical Phenotype	References
*FANCA*	c.3624C > T (p.S1208=) splicing variant	Delayed onset of hematological abnormalities Increased survival Reduced incidence of cancer Improved fertility	[29]
	c.4199G > A (p.R1400H)	Esophageal squamous cell carcinoma (age 51) Normal blood counts Preserved fertility	[30]
	c.2738A > C (p.H913P) c.2852G > A (p.R951Q) c.2851C > T (p.R951W)	Late onset of mild cytopenia Defects in mitochondrial function Note: solid tumor incidence not reported likely due to the age of study participants (all children except one young adult)	[31]
*FANCB*	c.353T > C (p.F118S) c.986T > C (p.L329P) c.1435T > G (p.W479G) c.2027T > C (p.L676P) c.2249G > T (p.G750V)	Later onset cytopenia and longer survival than individuals with FANCB nonsense or truncating variants	[2]
*FANCC*	c.67delG (p.D23IfsTer23)	Mild FA phenotypes Note: solid tumor incidence not reported	[32,33,34]
*FANCD1/* *BRCA2*	5′ splicing variants	May confer a survival benefit	[35]
	c.1813dup c.7796 A > G	Breast cancer at 33 y/o Severe toxicity after chemotherapy No embryonal cancers or leukemia	[36]
	c.8524C > T (p.R2842C)	Premature ovarian insufficiency No FA phenotypes or cancer	[37]
*FANCS/* *BRCA1*	c.5096G > A (p.R1699Q)	Early onset breast cancer Mild FA-like features Significant toxicity from chemotherapy Negative chromosome breakage analysis	[38]
*FANCN/* *PALB2*	c.2586 + 1G > A (p.T839_K862del)	No severe congenital anomalies Normal CBC B-cell non-Hodgkin lymphoma	[39]
